# Vitality, viability, long-term clonogenic survival, cytotoxicity, cytostasis and lethality: what do they mean when testing new investigational oncology drugs?

**DOI:** 10.1007/s12672-023-00857-2

**Published:** 2024-01-05

**Authors:** Benjamin N. Forgie, Rewati Prakash, Alicia A. Goyeneche, Carlos M. Telleria

**Affiliations:** 1 Experimental Pathology Unit, Department of Pathology, Faculty of Medicine and Health Sciences, McGill University, Montreal, QC Canada; 2 Cancer Research Program, Research Institute, McGill University Health Centre, Montreal, QC Canada

**Keywords:** Viability, Vitality, Cytotoxicity, Cytostasis, Lethality, Clonogenicity

## Abstract

In the field of experimental therapeutics for oncology purposes researchers are continuously evaluating the toxicity of novel treatment approaches against cancer cells. Within this topic of research, it is highly critical to define parameters of toxicity that denote when cancer cells are perturbed in their functionality by a new investigational drug. As the goal for these approaches is to achieve cellular demise, then what approaches to use and what do they mean in terms of assessing such cell death is of critical importance. In this comment article we highlight the definition of vitality and differentiate it from viability, and further define clonogenic survival in a chronic fashion. Additionally, we highly recommend the use of the term cytotoxicity as a general descriptor indicating toxicity towards a cell, but within that we encourage to sub-classify it as either cytostasis (i.e., when a treatment does not allow a cell to grow but it does not kill it either), or lethality (when a cell dies in response to the treatment). A more precise use of these terms should help advance the field of experimental therapeutics in oncology towards better defining the mechanisms of action of novel investigational drugs.

## Introduction

Cell death is a key goal to achieve when testing the efficacy of new investigational drugs to treating cancer. However, treatments can achieve many outcomes that relate to cell death but that may not be precisely indicating demise. For instance, in response to a toxic agent, a cell may interrupt its capacity to grow by undergoing transient cell cycle arrest or acquire a senescence phenotype. Another toxic mechanism is that the cell indeed undergoes a dying process that culminates with its demise (what we can call lethality or cell death). Another possibility is that in response to the toxic chemical, the cell does not immediately die; it may survive but it loses its capacity to reproduce, meaning that it will not grow despite providing proper conditions of space, nutrients, and oxygen in the absence of the initial drug. Such impairment of the reproductive capacity of a cell that is not dead represents another mechanism of chronic toxicity to consider when evaluating the mechanism of action of novel cytotoxic drugs. Here we define with precision several terms that are used to denote cellular toxicity that must not be used interchangeably.

## Definition of controversial terms

### Vitality

Vitality is a term that is rarely used in the field of experimental therapeutics. However, it is a parameter that is often wrongly assessed and interpreted as representing viability. We suggest that vitality be defined as the metabolic health of the cell. This is the case when using the popular proliferation assays based on metabolic activities (revisited in [1]). These assays detect either mitochondrial enzyme activity or cellular metabolism based on the activation of cytoplasmic enzymes. Using these assays as markers of cytotoxicity is not correct as they only assess the wellbeing of the cell. Cells may be affected in some of their metabolic functions but that does not mean they will die in response to the treatment in question. In these cases, the term vitality better characterizes these cellular behaviors. Therefore, a cell with metabolic impairment is a cell without optimal health or wellbeing but does not represent whatsoever a cell committed to die. A definition of vitality in terms of the “physiological state” of the cell was clearly described in yeast and differentiated from the term viability which instead was termed as the “percentage of live cells in a whole population” [2].

Assays for assessing vitality can be categorized based on the method of detection; these are colorimetric, fluorometric, and luminometric–based assays. The colorimetric MTT, XTT, MTS, and WST assays are well known as simple, rapid, and inexpensive methods for assessing the metabolic capacity of cells [3, 4]. These assays function by measuring the reduction of tetrazolium salts by cellular NADPH-dependent oxidoreductase enzymes. Healthy cells with higher metabolic activity will reduce the tetrazolium salts into formazan crystals, the concentration of which can be measured spectrophotometrically. Damaged and dying cells will have reduced enzymatic capacity resulting in lower production of formazan. Popular fluorometric methods for assessing cell vitality include Calcein-AM, Alamar blue, and 5-CFDA-AM assays [5–7]. These initially non-fluorescent cell permeable dyes can pass through the membranes of live cells and be enzymatically converted into their fluorescent states. Specifically, Calcein-AM and 5-CFDA-AM are hydrolyzed by non-specific cytoplasmic esterases, while Alamar blue is reduced by various cellular reductases. These fluorescent signals can then be measured using fluorescence microscopy or fluorometers. In this way, healthy, live cells will fluoresce strongly while damaged cells with decreased enzymatic activity will display weaker fluorescence. Finally, luminometric methods, such as ATP assays, are another manner of measuring cell vitality [8]. ATP is the primary source of energy within cells, and thus, changes in ATP levels can reflect the health of the cell. The enzyme luciferase converts luciferin into oxyluciferin in an ATP dependent reaction. Therefore, the strength of the luminescent signal is proportional to the amount of ATP within the cell, with healthy live cells producing a stronger signal, and metabolically inactive or dying cells producing a weaker signal. Thus, assays for assessing the metabolic capacity of cells, such as those described above, should not be used when examining viability. This is because assays that inspect metabolic capacity and cellular respiration are unable to discriminate between dead cells and cells that simply have a reduced enzymatic capacity following treatment yet are still alive.

## Viability

The term viability specifically refers to cells with compromised plasma membrane integrity. To properly assess viability, methods that evaluate the integrity of the plasma membrane should be used instead of the commonly used metabolic assays described above. A common method for determining cell viability is using exclusion dyes, such as trypan blue [9], which only enters cells that have compromised plasma membranes. Under a light microscope, live cells with intact membranes will remain clear, whereas dead cells will take up the dye and appear blue. Similarly, fluorescent exclusion dyes can be used for flow cytometry and immunofluorescence to distinguish between viable and non-viable cells. For example, membrane impermeable dyes such as propidium iodide (PI) and 7-Aminoactinomycin D (7-AAD) can enter dead cells, bind to DNA, and fluoresce [10, 11]. Finally, a common method of assessing cell membrane integrity is through the lactate dehydrogenase (LDH) assay [12]. LDH is a cytoplasmic enzyme that is released into the cell culture media following membrane permeabilization during cell death. LDH mediates the conversion of lactate to pyruvate, in the process generating NADH, which can then be used in the reduction of a tetrazolium salt to formazan. This formazan salt can be measured colorimetrically using a spectrophotometer. As LDH is only released from cells with damaged membranes, only dead and dying cells will result in formazan production. We believe that using membrane integrity to define viability provides a more stringent requirement for cells to be considered dead. For example, cases of early apoptosis do not display decreased membrane integrity and as such we would still consider these cells viable, even though these cells may be undergoing early stages of the cell death process or in other words “dying,” but they are not yet dead. Only when the plasma membrane has lost its integrity can we claim the cell is truly dead.

## Traditional clonogenic survival versus long-term clonogenic survival

One way of testing the reproductive capacity of a cancer cell culture in response to an investigational drug is the use of a clonogenic survival assay. However, there are different manners of performing this study. The most common modality is to study the clonality of the cells by culturing them away from one another, providing nutrients and the treatment in question. Cells are seeded at sufficient distance from one another that if they multiply it is due to their intrinsic individual clonogenic capacity. When these dispersed cells are cultured in the presence of chemotherapeutic agents, if the agents are efficient, they will prevent either cellular division what would result in smaller colonies upon cultivation for a prolonged period or induce cell death. When a compound is tested in this manner, one calculates the survival fraction, which means the number of colonies formed divided by the number of seeded cells times 100% [13]. We propose a more common use of another less popular modality of clonogenic assay, which we term “long-term” clonogenic survival assay. This is defined by plating the assay after cultures have been treated previously with the drug in question but are no longer in the presence of such drug. Thus, the drug will have short-term toxicity in the culture, but it may have residual toxicity. Such residual toxicity can be evaluated using clonogenic survival assays done in a different manner. In this case the seeding cells are those that are still alive after the acute treatment. By seeding them as a clonogenic assay we test the long-term toxicity of the drug used in the original culture (see examples from our laboratory in [14–18]). The cells may not have died in a short term (say 24, 48 h), but the drug had a residual toxicity that is manifested in the loss of the clonogenicity of such cells. The main difference between the traditional clonogenic survival assay and the long-term clonogenic survival assay, is that in the traditional assay the clones grow in the presence of the testing drug, whereas in the “long-term” the clones grow in the presence of drug-fee media. Thus, we suggest that a better testing of the toxicity of a new drug is by assessing short-term toxicity using a viability assay and utilize remaining live cells after exposure to the drug to seed a long-term clonogenic survival assay in the absence of the drug to thus account for the global toxicity of the therapy under study.

### Cytotoxicity, cytostasis and lethality

A final comment on the nomenclature of toxicity is the vague nature of the term. The term toxicity should be used to denote that a drug is detrimental to the functionality of a cell. However, this can have a broad range of implications; we can say that a drug is toxic because it affects the metabolic balance of the cells, because it kills the cells, or because it does not kill the cells but impairs their capacity to reproduce. Therefore, we believe it is more useful to use the term *cytotoxicity* as a manifestation of an undefined toxicity to the cells. However, if there is evidence that the drug under study blocks the long-term clonogenic capacity of the cells, we can use the term *cytostasis*. On the other hand, when there is proof that cells die in response to treatment, then we can confirm that the drug in question is *lethal*.

## Conclusions

The concept of cell death and the ability to assess cell death is crucial for the field of pharmaceuticals and drug testing. Therefore, it is critical that the terminology being used to describe cell death accurately reflects the fate of the cells. In this commentary we expanded the nomenclature surrounding cell death to include the lesser-known term, vitality, alongside viability. Vitality provides flexibility when discussing cellular toxicity, as it considers the health of the cell as described by metabolic capacity and cellular respiration. Viability, however, should refer only to cases of cell death specifically denoted by damaged or compromised plasma membranes. Assays that assess vitality or viability share a common disadvantage that is the oversight of long-term toxicity. Drugs that do not strongly affect vitality or viability in short-term studies may have significant effects on the long-term reproductive capacity of the cells. Clonogenic survival assays are methods that can be used to assess the reproductive capacity of drug-treated cells. Here, we suggest the use of a “long-term” clonogenic survival, which is better able to assess the residual toxicity of the drug when compared with the traditional clonogenic survival assay. A potential toxic drug should not be discarded as a candidate therapeutic agent against cancer using methods that evaluate vitality or viability only. They can have a long-lasting toxic effect that is only manifested in their long-term reproductive capacity tested in long-term clonogenic survival assays. Finally, when discussing drug toxicity, it is important to note that the term cytotoxicity is an umbrella term which encompasses the more specific descriptors: cytostasis and lethality.

## Data Availability

The detailed information provided in this manuscript is openly available to the scientific community upon request.
